# Supplementary material for: A Novel Tandem-Tag Purification Strategy for Challenging Disordered Proteins
Source: Biomolecules. 2022 Oct 26;12(11):1566. doi: 10.3390/biom12111566 (PMC9687501; doi:10.3390/biom12111566)
Supplement: Supplementary file 1 [file biomolecules-12-01566-s001.zip › biomolecules-1966787-supplementary.pdf]

5. Supplementary Materials:

I. Gel pictures:

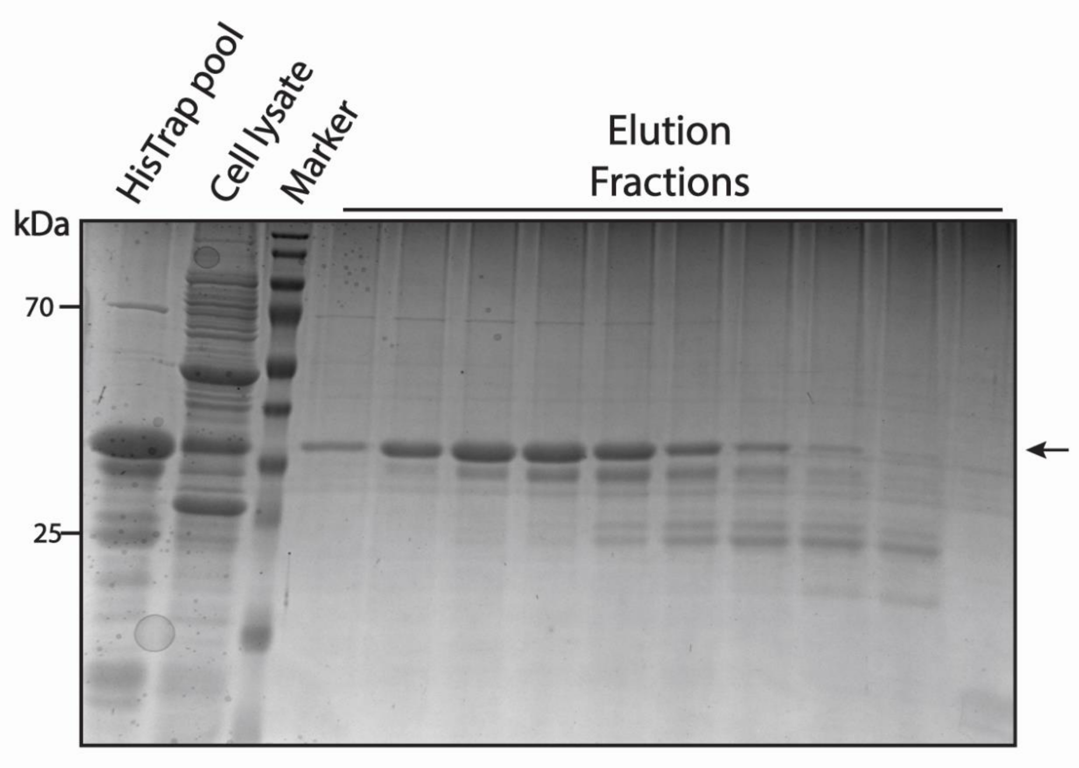

Figure S1.: Purification of pSUMO-AF1(only N-tag). After the Histrap the sample is degraded which cannot be completely separated by SEC using as indicated by the elution fractions. The black arrow indicates full-length AF1 after cleavage of the tag.

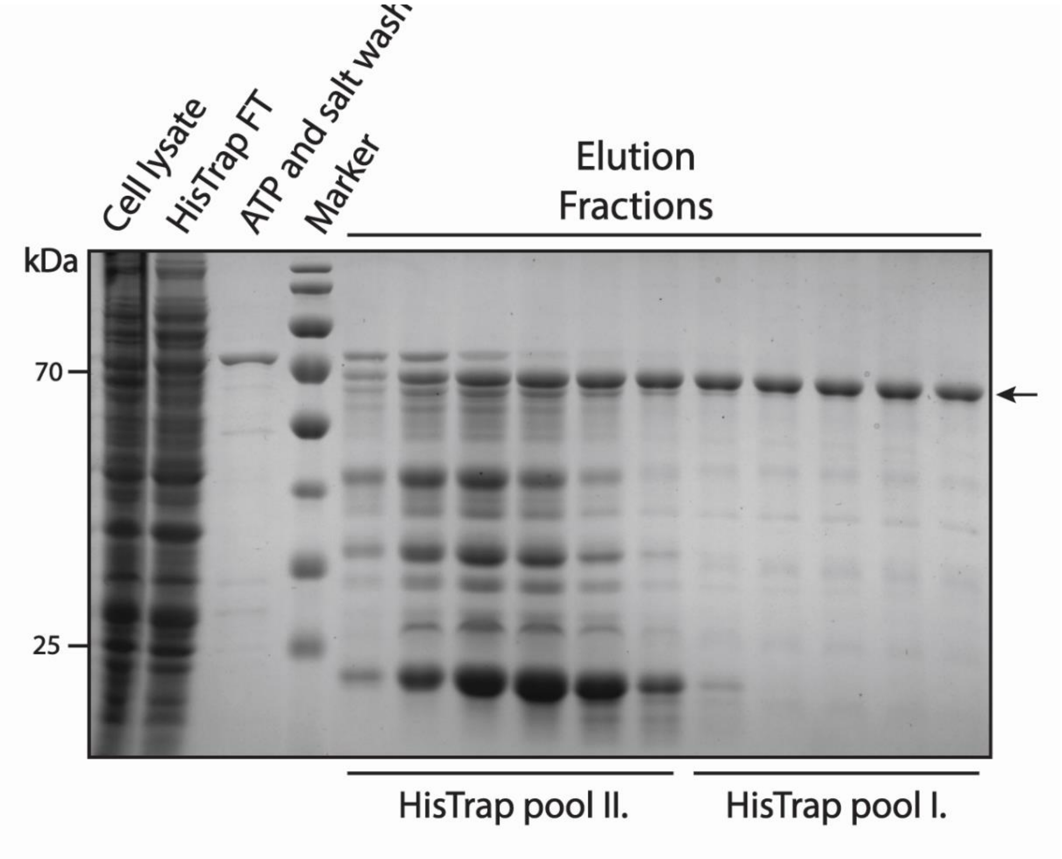

Figure S2.: HisTrap purification of pSUMO-AF1. In the elution fractions there are a clean region of fractions (HisTrap pool I) and a more degraded region (HisTrap pool II). Separately, these were further processed. The black arrow indicates the full-length AF1 before cleaving the tags.

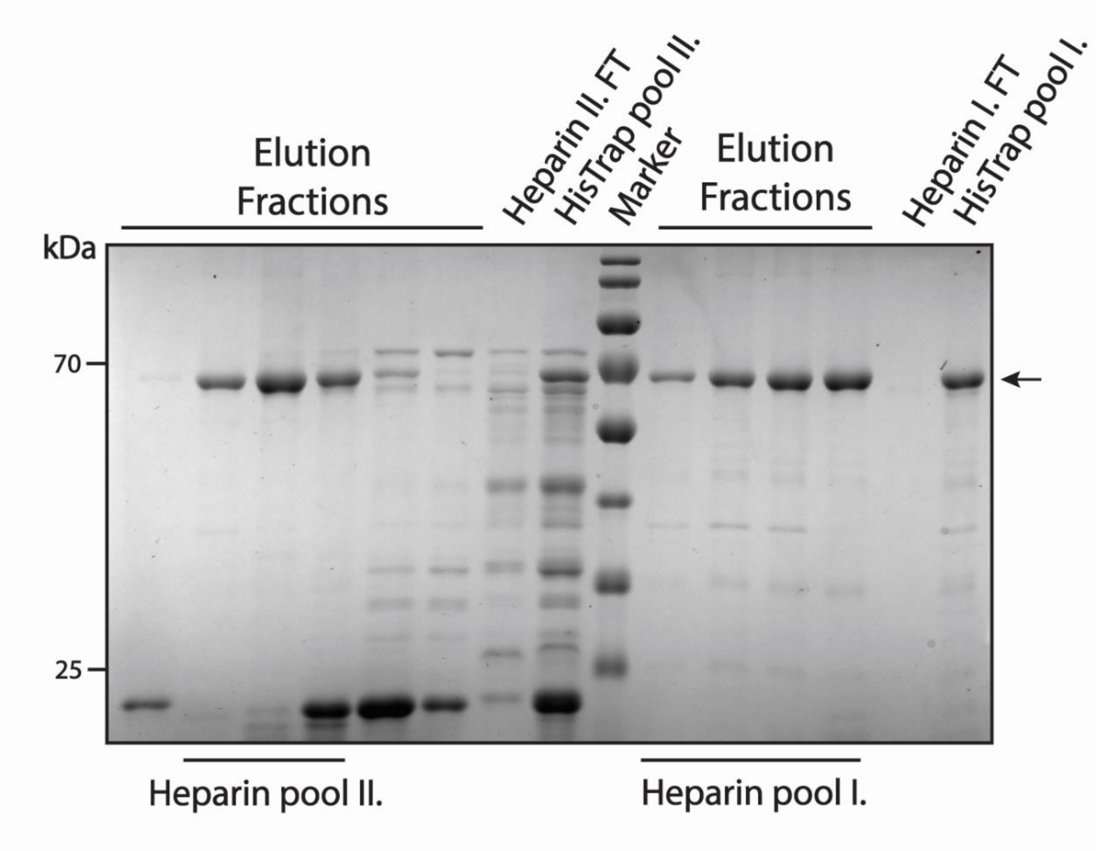

Figure S3.: Heparin purification of AF1. The two different pools from HisTrap (HisTrap pool I and II) were processed separately as Heparin pool I and Heparin pool II. The black arrow indicates the full-length AF1 before cleaving the tags.

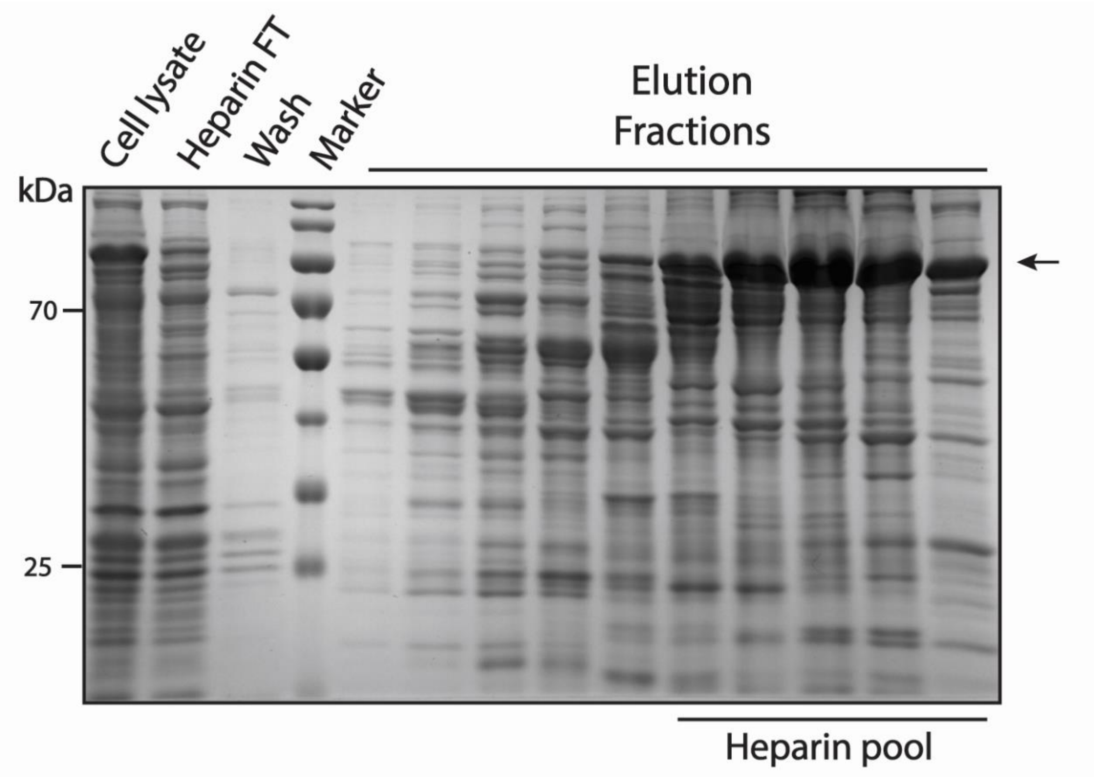

Figure S4.: Heparin purification of pSUMO-Tau-441. The indicated fractions (Heparin pool) were pooled together for the next chromatographic step. The black arrow indicates the full-length Tau-441 before cleaving the tags.

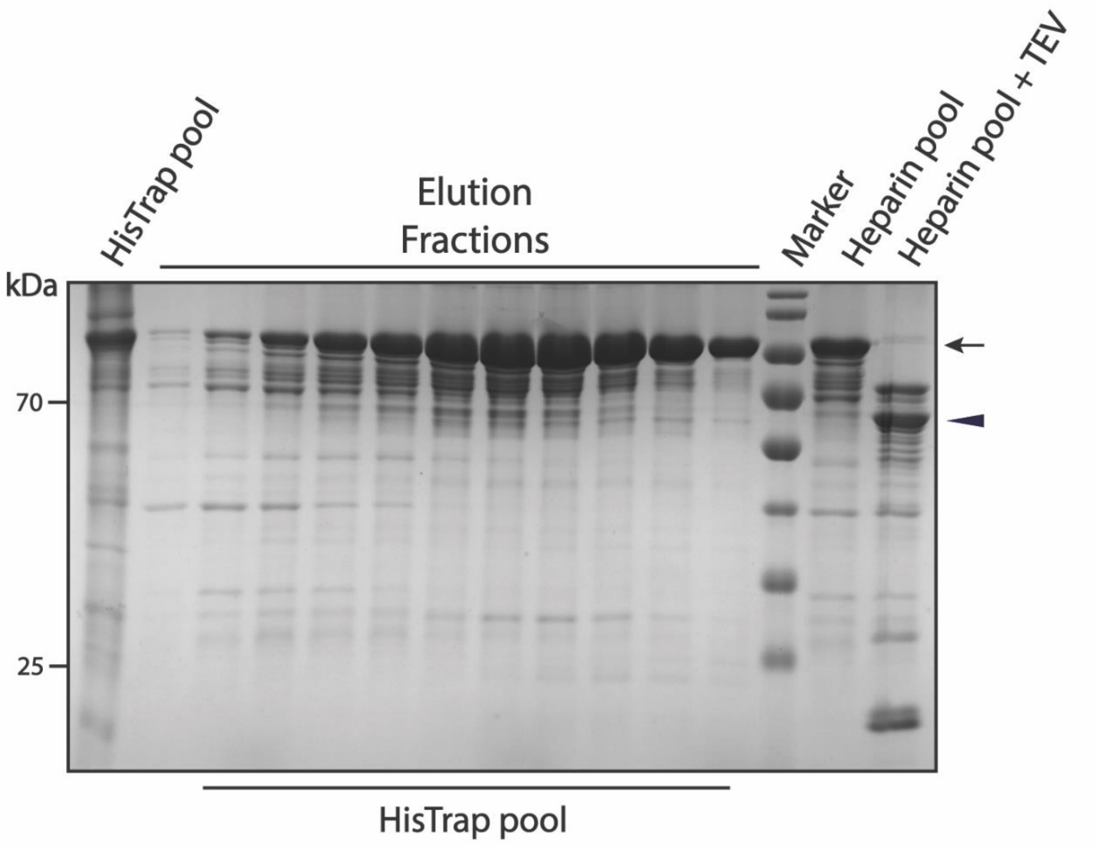

Figure S5.: HisTrap purification of pSUMO-Tau-441. The indicated fractions (HisTrap pool) were pooled for cleavage and subsequent chromatography. The black arrow indicates intact Tau-441 having both affinity tags, while the black triangle indicates the cleaved final product.

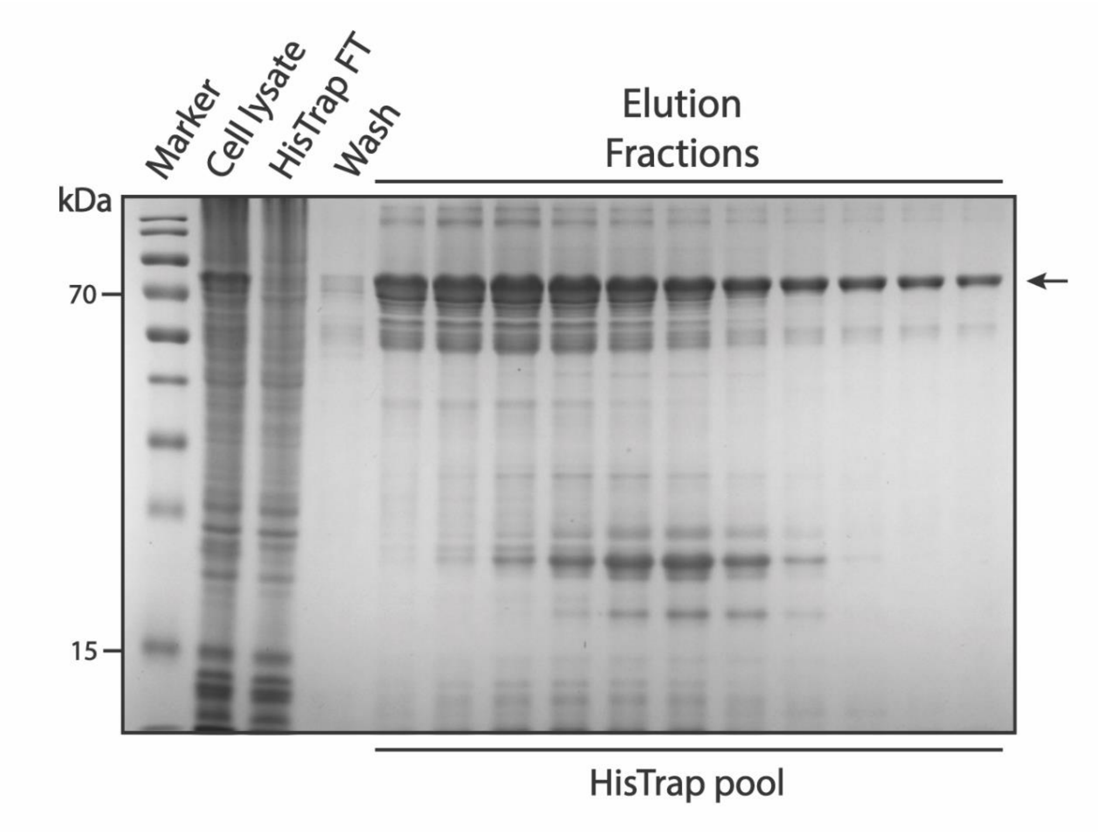

Figure S6.: HisTrap purification of pSUMO-Tau-NTMT. The full-length protein was the most dominant protein species in all elution fractions. Therefore, all elution fractions were pooled together and prepared for Heparin chromatographic separation. The black arrow indicates full-length Tau-NTMT with both affinity tags.

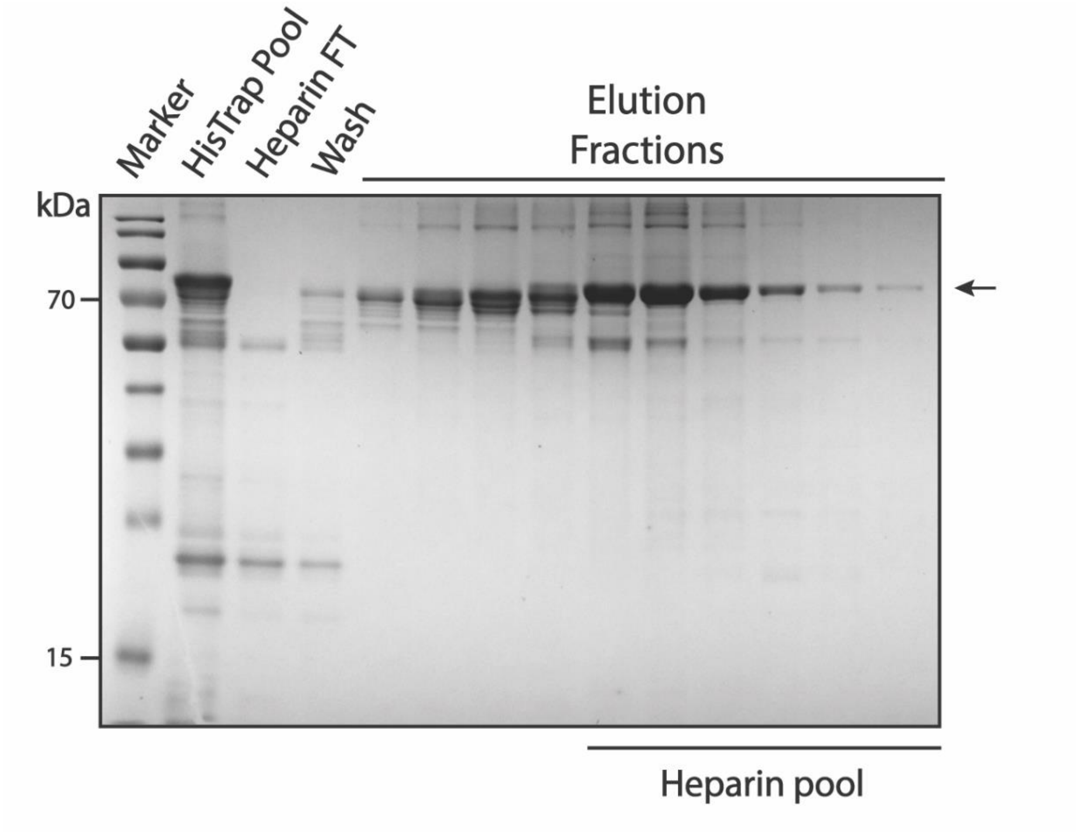

Figure S7.: Heparin purification of pSUMO-Tau-NTMT. Majority of the full-length protein eluted in later fractions. These were pooled together for dialysis and TEV digestion, prior to Reverse HisTrap purification. The black arrow indicates full-length Tau-NTMT with both affinity tags.

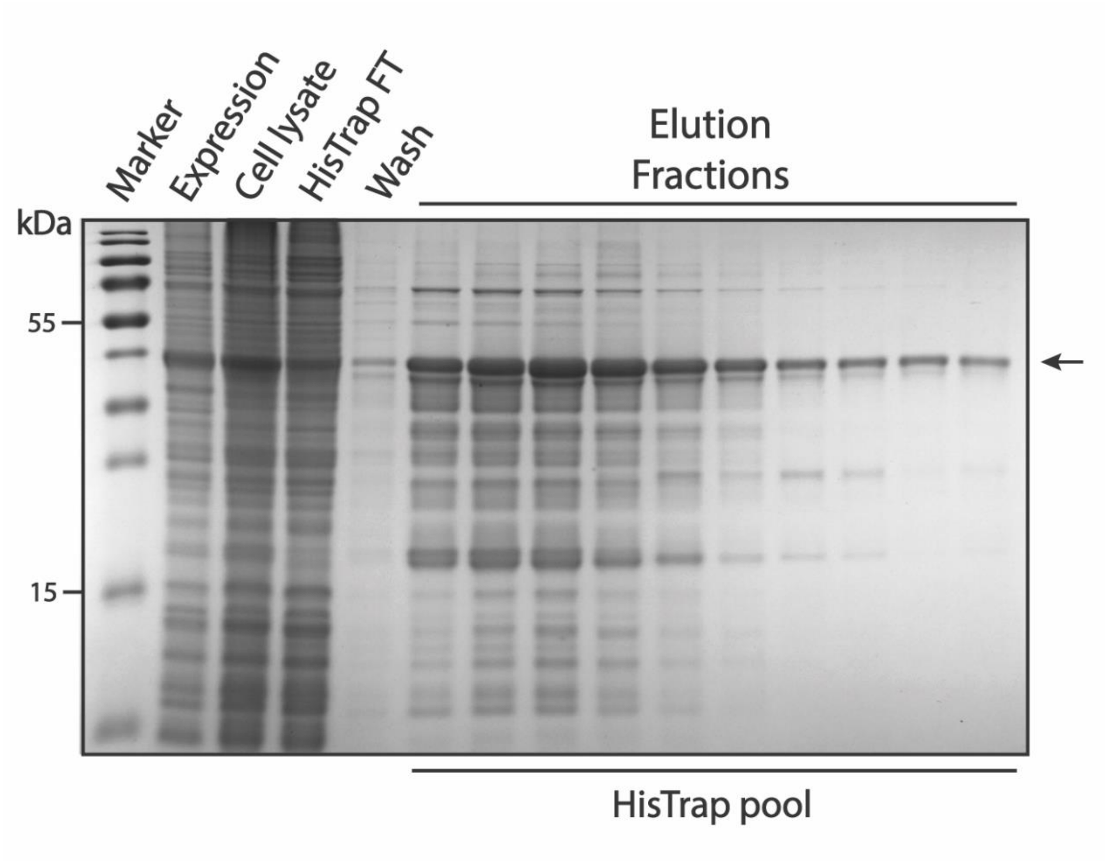

Figure S8.: HisTrap purification of pSUMO-Tau-MTBR. The full-length protein was the most dominant protein species in all elution fractions. Therefore, all elution fractions were pooled together and prepared for Heparin chromatographic separation. The black arrow indicates full-length Tau-MTBR with both affinity tags.

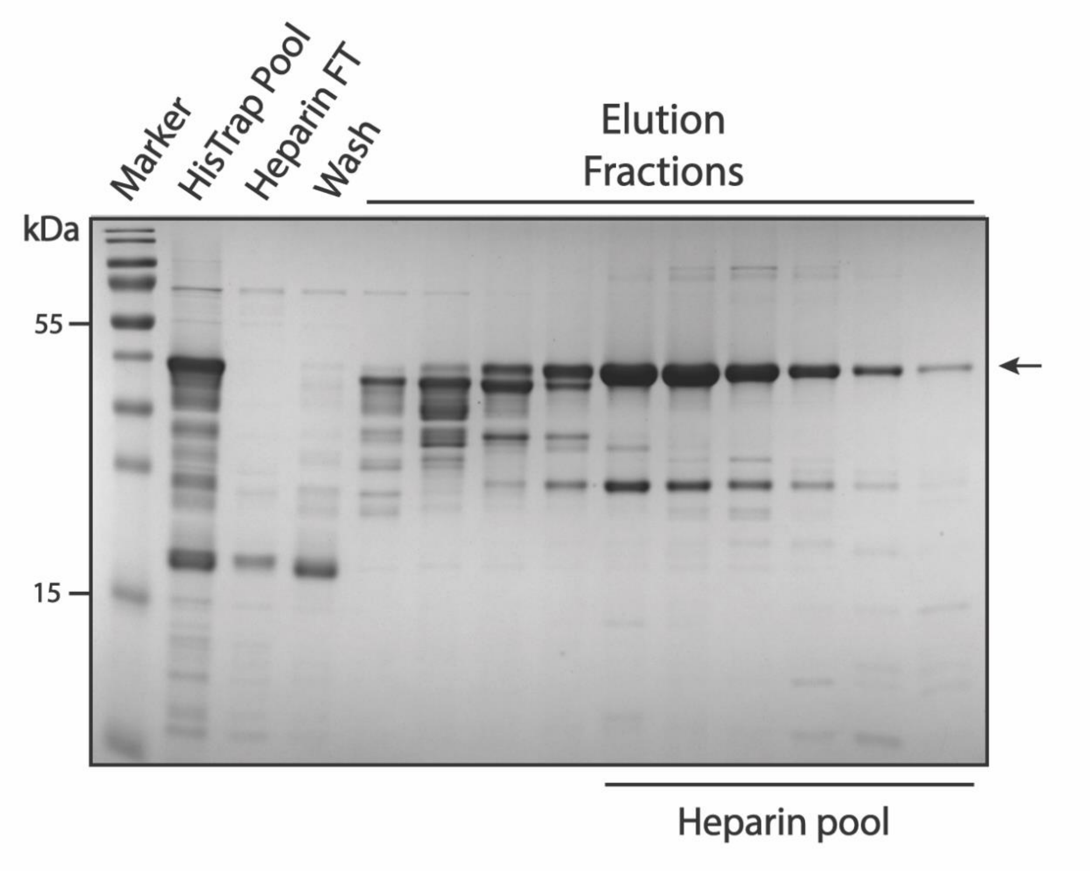

Figure S9.: Heparin purification of pSUMO-Tau-MTBR. Majority of the full-length protein eluted in later fractions. These were pooled together for dialysis and TEV digestion, prior to Reverse HisTrap purification. The black arrow indicates full-length Tau-MTBR with both affinity tags.

II. MS Analysis:

1. MS analysis of p-SUMO-AF1:

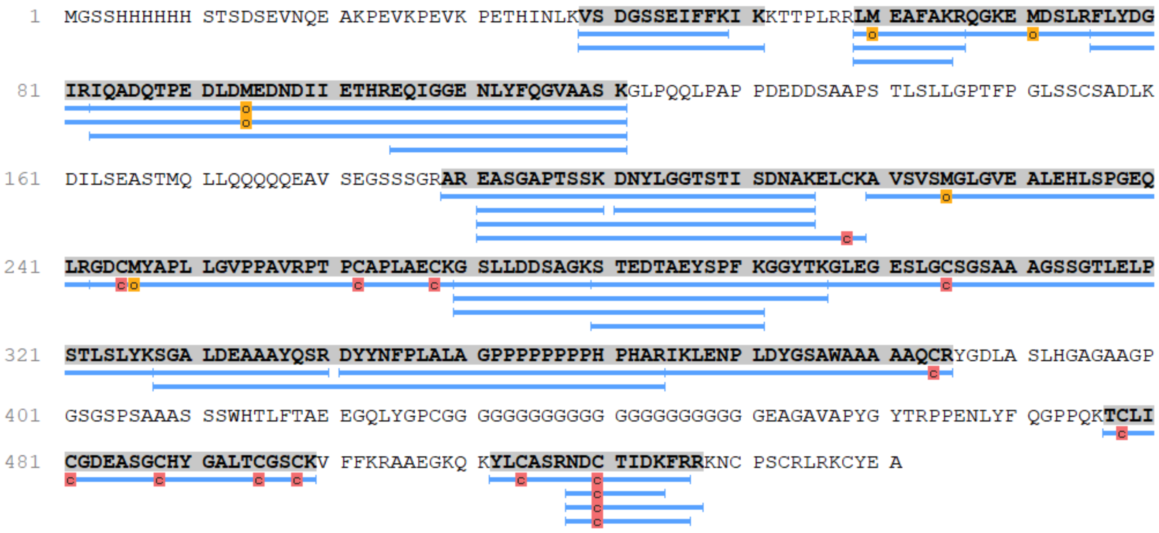

Figure S10.: Alignment of identified peptides from the uncleaved sample (before TEV cleavage) to the sequence uncleaved product (AF1 with both tags), indicating that AF1 is present with both tags. The N-terminal 6xHis-SUMO-tag with the TEV cleavage site starts from residue 1 to residue 116, while the C-terminal DBD with the TEV cleavage site starts from residue 466 to residue 541. Oxidised methionines and carbamidomethylated cysteines are peptide modifications highlighted with O-symbol (orange) and C-symbol (red), respectively.

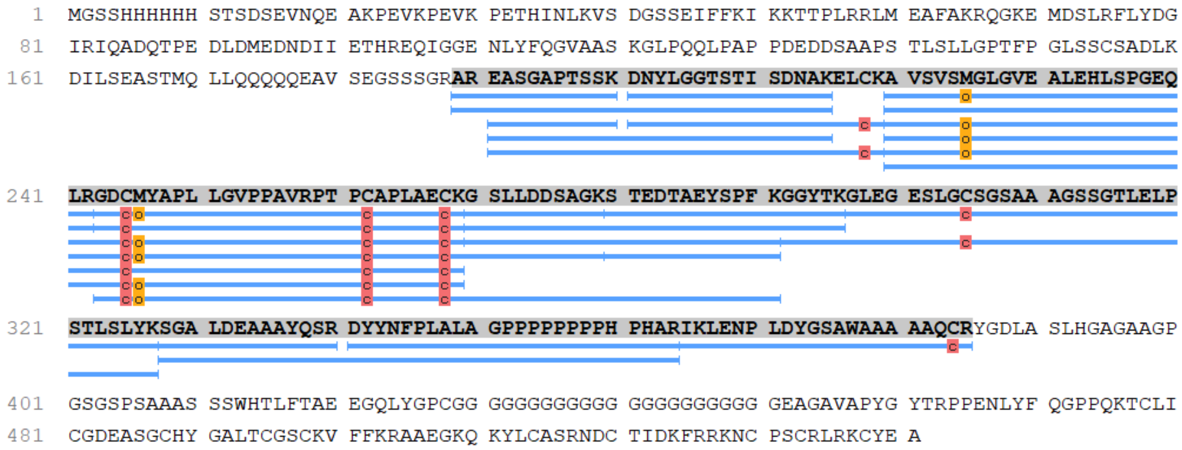

Figure S11.: Alignment of identified peptides from the cleaved sample (final product) to the sequence of uncleaved product (AF1 with both tags), indicating that both tags have been removed. The N-terminal 6xHis-SUMO-tag with the TEV cleavage site starts from residue 1 to residue 116, while the C-terminal DBD with the TEV cleavage site starts from residue 466 to residue 541. Oxidised methionines and carbamidomethylated cysteines are peptide modifications highlighted with O-symbol (orange) and C-symbol (red), respectively.

2. MS analysis of p-SUMO-Tau-441:

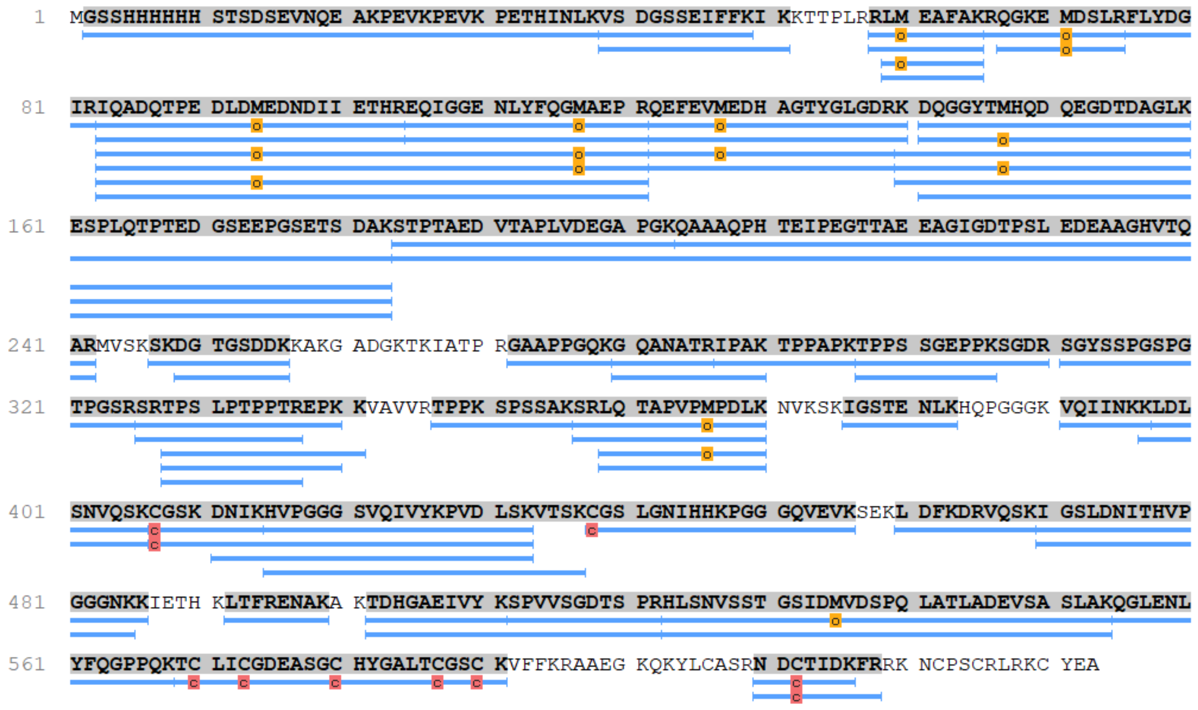

Figure S12.: Alignment of identified peptides from the uncleaved sample (before TEV cleavage) to the sequence of uncleaved full-length construct (Tau-441 with both tags), indicating that Tau-441 is present with both tags. The N-terminal 6xHis-SUMO-tag with the TEV cleavage site starts from residue 1 to residue 116, while the C-terminal DBD with the TEV cleavage site starts from residue 558 to residue 663. Oxidised methionines and carbamidomethylated cysteines are peptide modifications highlighted with O-symbol (orange) and C-symbol (red), respectively.

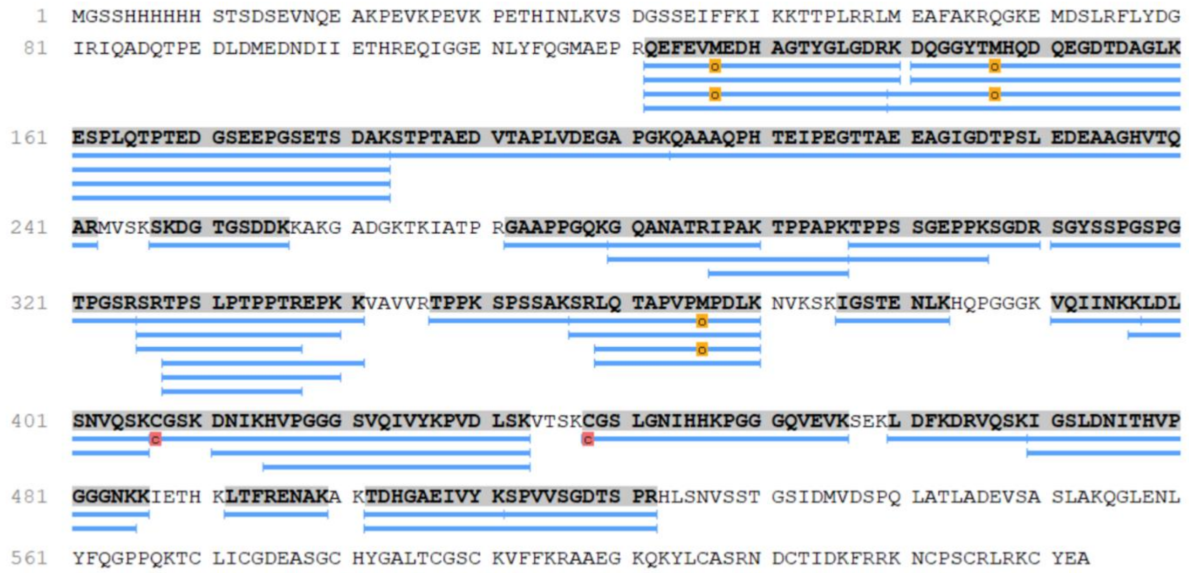

Figure S13.: Alignment of identified peptides from the cleaved sample (final product) to the sequence of uncleaved full-length construct (Tau-441 with both tags), indicating that both tags have been removed. The N-terminal 6xHis-SUMO-tag with the TEV cleavage site starts from residue 1 to residue 116, while the C-terminal DBD with the TEV cleavage site starts from residue 558 to residue 663. Oxidised methionines and carbamidomethylated cysteines are peptide modifications highlighted with O-symbol (orange) and C-symbol (red), respectively.

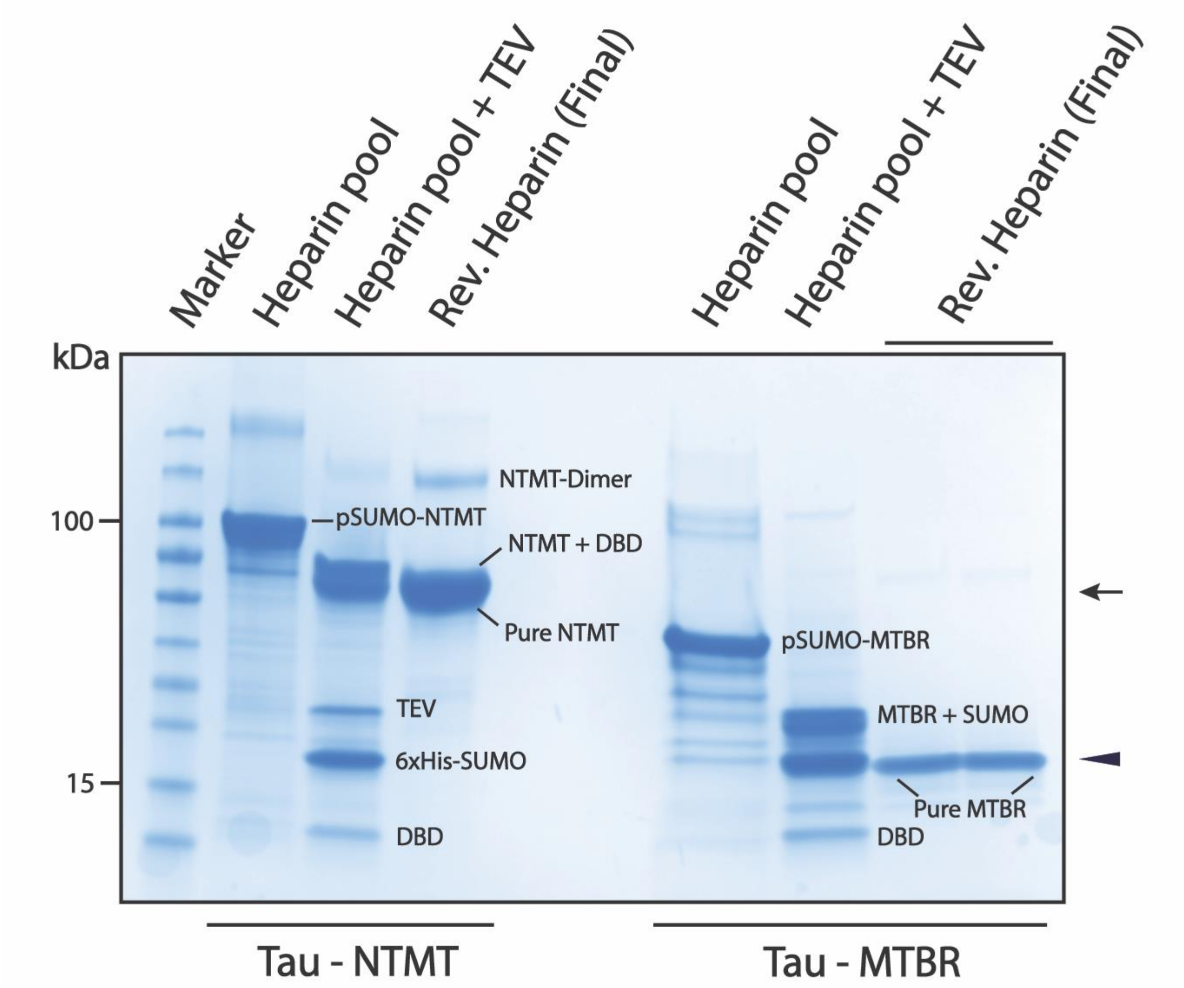

Figure S14.: SDS-PAGE Gel highlighting bands that were extracted, analysed and confirmed by MS. The black arrow indicates purified Tau-NTMT final product, while the black triangle indicates purified Tau-MTBR final product.

3. **MS analysis of pSUMO-Tau-NTMT:**

pSUMO-NTMT band:

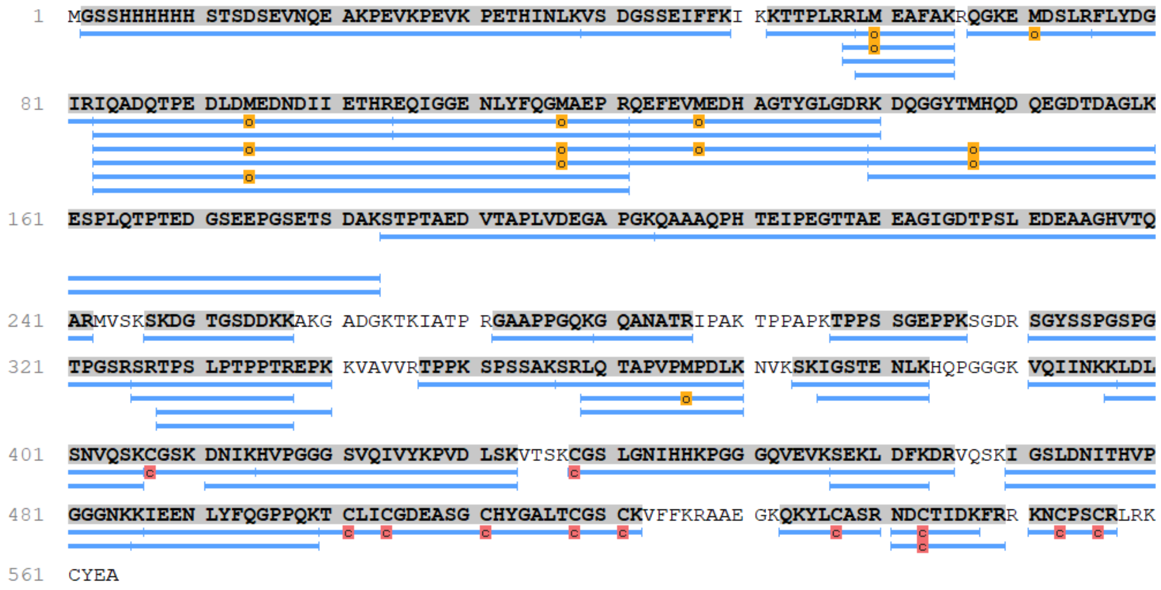

Figure S15.: Alignment of identified peptides from uncleaved sample (before TEV cleavage) to the sequence of uncleaved full-length construct (Tau-NTMT with both tags), indicating that both tags are present before TEV cleavage. The N-terminal 6xHis-SUMO-tag with the TEV cleavage site starts from residue 1 to residue 116, while the C-terminal DBD with the TEV cleavage site starts from residue 489 to residue 564. Oxidised methionines and carbamidomethylated cysteines are peptide modifications highlighted with O-symbol (orange) and C-symbol (red), respectively.

NTMT+DBD band:

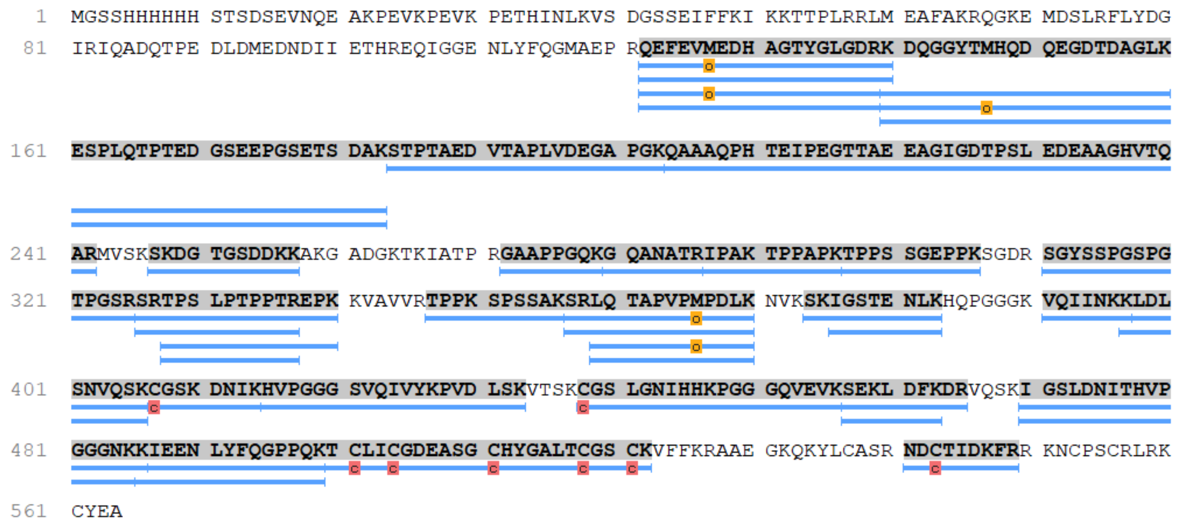

Figure S16.: Alignment of identified peptides from partially cleaved sample (after TEV cleavage) to the sequence of uncleaved full-length construct (Tau-NTMT with both tags), indicating that only the 6xHis-SUMO-tag has been removed. The N-terminal 6xHis-SUMO-tag with the TEV cleavage site starts from residue 1 to residue 116, while the C-terminal DBD with the TEV cleavage site starts from residue 489 to residue 564. Oxidised methionines and carbamidomethylated cysteines are peptide modifications highlighted with O-symbol (orange) and C-symbol (red), respectively.

Pure NTMT band (final product):

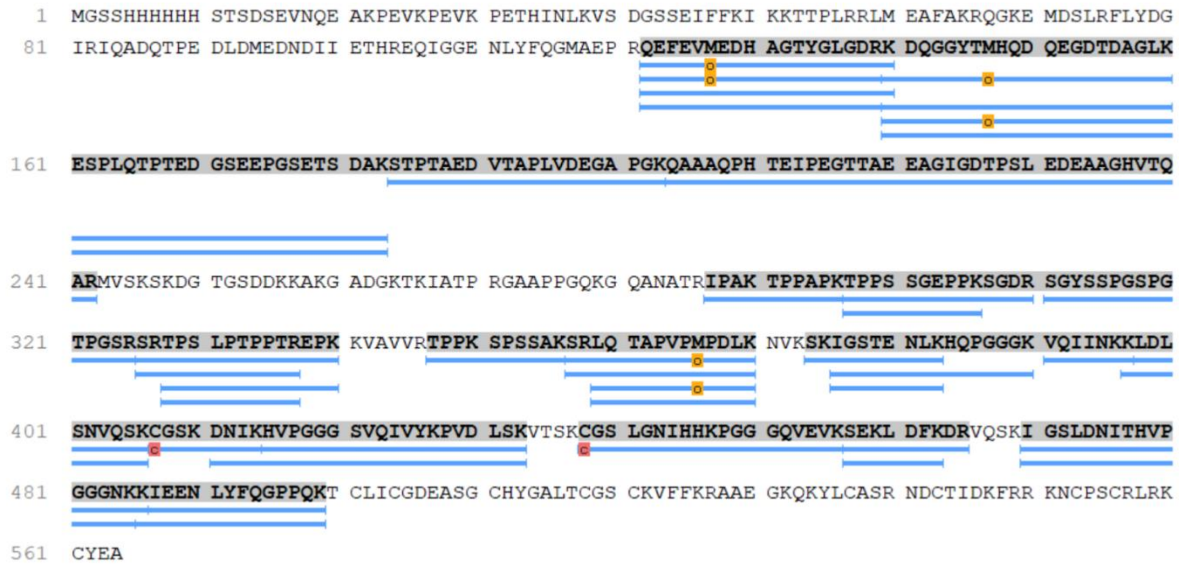

Figure S17.: Alignment of identified peptides from the cleaved sample (final product) to the sequence of uncleaved full-length construct (Tau-NTMT with both tags), indicating that both tags have been removed. The N-terminal 6xHis-SUMO-tag with the TEV cleavage site starts from residue 1 to residue 116, while the C-terminal DBD with the TEV cleavage site starts from residue 489 to residue 564. Oxidised methionines and carbamidomethylated cysteines are peptide modifications highlighted with O-symbol (orange) and C-symbol (red), respectively.

NTMT-Dimer band:

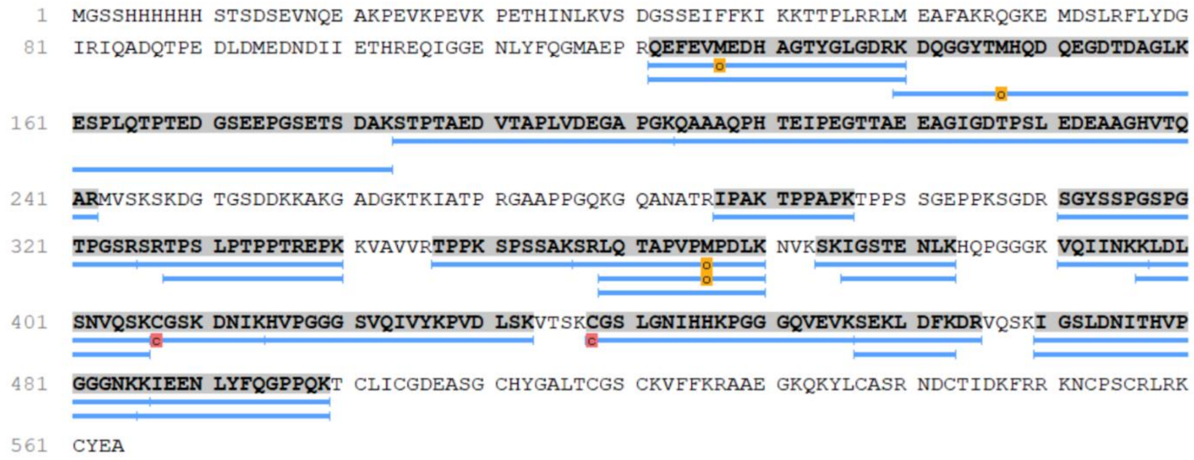

Figure S18.: Alignment of identified peptides from high-molecular-weight (HMW) bands in Tau-NTMT final product (NTMT-Dimer, ~120 kDa band) to the sequence of uncleaved full-length construct (Tau-NTMT with both tags), indicating that the bands were made up of pure Tau-NTMT dimers. The N-terminal 6xHis-SUMO-tag with the TEV cleavage site starts from residue 1 to residue 116, while the C-terminal DBD with the TEV cleavage site starts from residue 489 to residue 564. Oxidised methionines and carbamidomethylated cysteines are peptide modifications highlighted with O-symbol (orange) and C-symbol (red), respectively.

4. MS analysis of pSUMO-Tau-MTBR:

pSUMO-MTBR band:

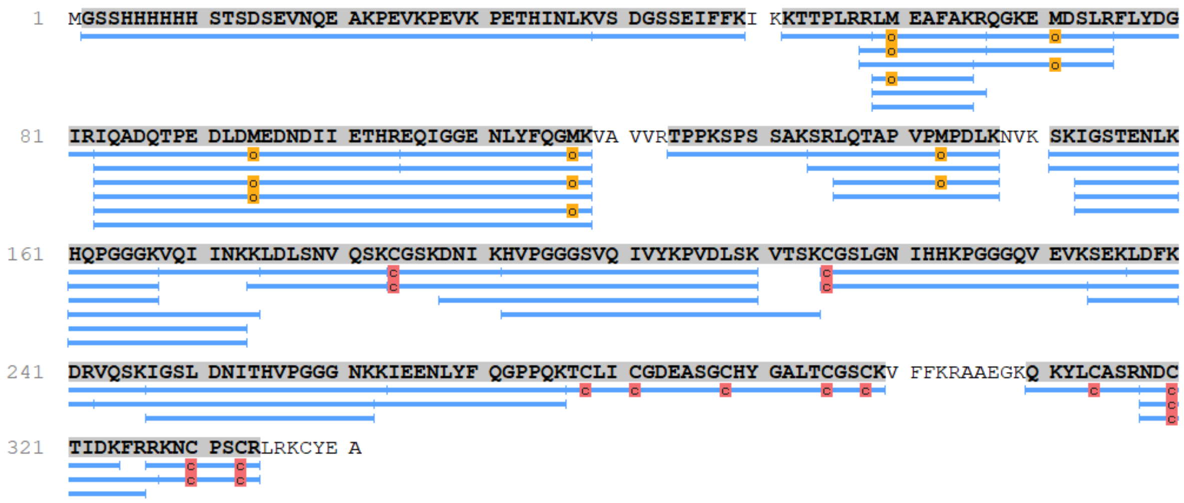

Figure S19.: Alignment of identified peptides from the uncleaved sample (before TEV cleavage) to the sequence of uncleaved full-length construct (Tau-MTBR with both tags), indicating that Tau-441 is present with both tags. The N-terminal 6xHis-SUMO-tag with the TEV cleavage site starts from residue 1 to residue 116, while the C-terminal DBD with the TEV cleavage site starts from residue 266 to residue 341. Oxidised methionines and carbamidomethylated cysteines are peptide modifications highlighted with O-symbol (orange) and C-symbol (red), respectively.

MTBR+SUMO band:

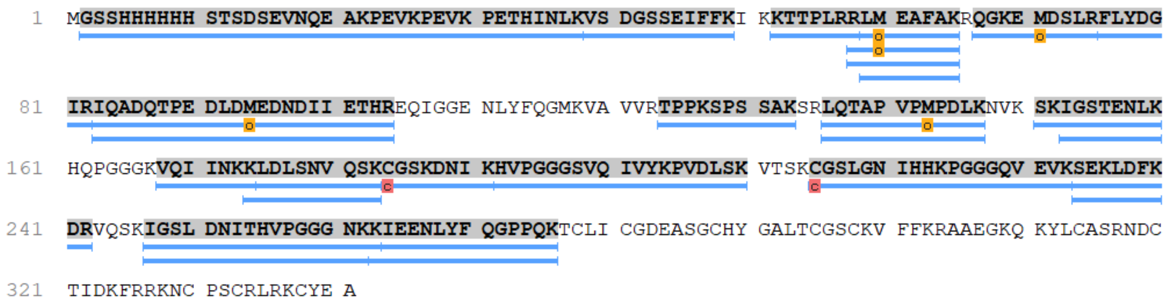

Figure S20.: Alignment of identified peptides from the partially cleaved sample (after TEV cleavage) to the sequence of uncleaved full-length construct (Tau-MTBR with both tags), indicating that Tau-441 still has the N-terminal 6xHis-SUMO-tag. The N-terminal 6xHis-SUMO-tag with the TEV cleavage site starts from residue 1 to residue 116, while the C-terminal DBD with the TEV cleavage site starts from residue 266 to residue 341. Oxidised methionines and carbamidomethylated cysteines are peptide modifications highlighted with O-symbol (orange) and C-symbol (red), respectively.

MTBR band (final product):

```
  1  MGSSHHHHHH STSDSEVNQE AKPEVKPEVK PETHINLKVS DGSSEIFFKI KKTTPLRRLM EAFAKRQGKE MDSLRFLYDG
 81  IRIQADQTPE DLDMEDNDII ETHREQIGGE NLYFQGMKVA VVRTPPKSPS SAKSRLQTAP VPMPDLKNVK SKIGSTENLK
161  HQPGGGKVQI INKKLDLSNV QSKCGSKDNI KHVPGGGSVQ IVYKPVDLSK VTSKCGSLGN IHHKPGGGQV EVKSEKLDFK
241  DRVQSKIGSL DNITHVPGGG NKKIEENLYF QGPPQKTCLI CGDEASGCHY GALTCGSCKV FFKRAAEGKQ KYLCASRNDC
321  TIDKFRRKNC PSCRLRKCYE A
```

Figure S21.: Alignment of identified peptides from the cleaved sample (final product) to the sequence of uncleaved full-length construct (Tau-MTBR with both tags), indicating that both tags have been removed. The N-terminal 6xHis-SUMO-tag with the TEV cleavage site starts from residue 1 to residue 116, while the C-terminal DBD with the TEV cleavage site starts from residue 266 to residue 341. Oxidised methionines and carbamidomethylated cysteines are peptide modifications highlighted with O-symbol (orange) and C-symbol (red), respectively.
